# Reversion of AHRR Demethylation Is a Quantitative Biomarker of Smoking Cessation

**DOI:** 10.3389/fpsyt.2016.00055

**Published:** 2016-04-06

**Authors:** Robert Philibert, Nancy Hollenbeck, Eleanor Andersen, Shyheme McElroy, Scott Wilson, Kyra Vercande, Steven R. H. Beach, Terry Osborn, Meg Gerrard, Frederick X. Gibbons, Kai Wang

**Affiliations:** ^1^Department of Psychiatry, University of Iowa, Iowa City, IA, USA; ^2^Behavioral Diagnostics, Iowa City, IA, USA; ^3^Department of Internal Medicine, University of Iowa, Iowa City, IA, USA; ^4^Department of Psychology, Center for Family Research, University of Georgia, Athens, GA, USA; ^5^Department of Psychology, University of Connecticut, Storrs, CT, USA; ^6^Department of Biostatistics, College of Public Health, University of Iowa, Iowa City, IA, USA

**Keywords:** DNA methylation, epigenetics, aryl hydrocarbon receptor repressor, cg05575921, diagnostics, smoking cessation

## Abstract

Smoking is the largest preventable cause of morbidity and mortality in the world. Although there are effective pharmacologic and behavioral treatments for smoking cessation, our inability to objectively quantify smokers’ progress in decreasing smoking has been a barrier to both clinical and research efforts. In prior work, we and others have shown that DNA methylation at cg05575921, a CpG residue in the aryl hydrocarbon receptor repressor (AHRR), can be used to determine smoking status and infer cigarette consumption history. In this study, we serially assessed self-report and existing objective markers of cigarette consumption in 35 subjects undergoing smoking cessation therapy, then quantified DNA methylation at cg05575921 at study entry and three subsequent time points. Five subjects who reported serum cotinine and exhaled carbon monoxide verified smoking abstinence for the 3 months prior to study exit averaged a 5.9% increase in DNA methylation at cg05575921 (*p* < 0.004) over the 6-month study. Although the other 30 subjects did not achieve smoking cessation at the 6-month time point, their self-reported reduction of cigarette consumption (mean = 6 cigarettes/day) was associated with a 2.8% increase DNA methylation at cg05575921 (*p* < 0.05). Finally, a survey of subjects as they exited the study demonstrated strong support for the clinical use of epigenetic biomarkers. We conclude that AHRR methylation status is a quantifiable biomarker for progress in smoking cessation that could have substantial impact on both smoking cessation treatment and research.

## Introduction

Smoking is the largest cause of preventable morbidity and mortality in the United States. Each year, nearly a half-million Americans die secondary to the effects of smoking (1). Still, nearly one in every five US adults currently smoke (2). Currently, three pharmacological agents, bupropion, varenicline, and nicotine replacement therapy (NRT), are commonly used for smoking cessation (3). By themselves, each of these medications is modestly effective and recent clinical trials suggest that the combination of varenicline and NRT is most effective in achieving cessation (3, 4). Nevertheless, the efficacy of these treatments in actual clinical practice has been less than optimal (5).

Although many barriers to the effective implementation of these and smoking cessation interventions exist, one of the more difficult hurdles to overcome is our current inability to quantify decreases in smoking and the success of cessation therapy. In epidemiologic studies, self-report is generally accurate, however, in clinical populations, it is much less reliable (6–8). Currently, two biological methods are commonly used to determine the success of therapy and corroborate self-report: exhaled carbon monoxide (CO) and cotinine levels. Exhaled CO levels are perhaps the easiest assessments to perform. But this measure is only capable of detecting smoking in the past 3–4 h and is not useful in qualifying changes in smoking at these levels because it is relatively insensitive to light-to-moderate smoking (9, 10). By contrast, assessments of cotinine, which has a serum half-life of 15 h, are much more sensitive and can detect smoking in the past 48–72 h (11). However, because false positives can arise from other forms of tobacco consumption (second-hand smoke, e-cigarettes, and ironically, NRT use) its clinical utility in monitoring decreased smoking and abstinence is limited. In fact, since over one-fourth of all patients who successfully quit smoking using NRT remain on NRT for at least 1 year after smoking cessation (12), the efficacy of employing cotinine levels to guide smoking cessation in clinical settings is minimal.

The development of quantitative continuous dose–response measures of decreases in smoking in therapy could significantly advance smoking cessation efforts in much the same way that the introduction of hemoglobin A1C (HbA1c) levels to assess the need and effectiveness of diabetes management has revolutionized the treatment of Type 2 diabetes (T2DM) (13). The use of HbA1c assessment, which is a measurement of the level of the acetylation of hemoglobin by serum glucose, allows clinicians to not only diagnose T2DM but also objectively quantify the progress of diabetic therapy. The latter is particularly important because numerous studies have shown that patients, in particular those who are at the highest risk, do not accurately report treatment compliance (14). The same challenges confront clinicians dealing with smoking cessation, suggesting that if a similar tool for measuring smoking intensity could be developed, it is possible that clinicians could use that assessment to detect changes in smoking, and modify treatment strategies during therapy.

DNA methylation assessments may provide a tool that can accurately assess amount and changes in smoking status in order to track the trajectory of smoking initiation and cessation. Over the past 3 years, at least 20 studies have confirmed the initial findings that methylation at cg05575921, a CpG residue in the aryl hydrocarbon receptor repressor (AHRR), is the most sensitive indicator of smoking status at all levels of smoking (15, 16). In particular, in a recent clinical trial, methylation status at this locus was employed to classify the smoking status of adult subjects, and was shown to be extremely accurate with a receiver operating characteristic (ROC) area under the curve (AUC) of 0.99 (15). Whereas these and other studies clearly indicate that DNA methylation can be used to track changes in smoking, they have not addressed the question of whether DNA methylation could be used to guide smoking cessation therapy.

Several genome-wide studies, including work from Zeilinger and colleagues and Tsaprouni and colleagues have compared smokers’ and non-smokers’ methylation at specific loci and concluded that the smoking-induced DNA methylation signature reverts as a function of long-term abstinence with cg05575921 being one of the most prominent loci demonstrating reversion (17, 18). In addition, however, Zeilinger et al. estimated that the speed of that reversion was relatively slow, with a change of approximately 7% occurring over a course of 7 years. If this is correct, this would suggest that DNA methylation changes relatively slowly and could not be used for monitoring smoking cessation.

However, recent evidence has suggested that the speed of reversion of DNA methylation at cg05575921 may be significantly faster than that estimate. In our recent examination of alcoholic inpatients, those who smoked prior to admission but were either completely or partially deprived of cigarettes during their stay, averaged a 1.7% increase in CG05575921 over 25 days (19). Second, both the Zeilinger and Tsaprouni studies were cross-sectional studies that employed self-report without biochemical verification of smoking status. Since the reliability of retrospective recall of smoking cessation is poor (20) and their study design did not allow before and after comparisons of individual subjects, the speed of methylation reversion in Zeilinger and Tsaprouni reports may be an underestimate. In this study, we directly examine the relationship between cigarette consumption status and DNA methylation at cg05575921 in a cohort of subjects undergoing smoking cessation therapy under the direction of their personal physicians.

## Materials and Methods

All methods and procedures used in this study were approved by the University of Iowa Institutional Review Board. In brief, the subjects were recruited by direct advertising and word of mouth from University of Iowa affiliated clinical operations. Inclusion criteria for the screening of potential subjects for the study included the following: being a current active smoker who was getting ready to begin smoking cessation within 4 days of the intake appointment, and abstinence from any nicotine-containing product, including e-cigarettes. Please note that the rationale for exclusion of those subjects using other forms of nicotine-containing products was to allow the team to use cotinine assays to detect surreptitious smoking. Non-combustionable forms of tobacco consumption do not have an effect on cg05575921 levels (15, 21). Other exclusion criteria included use of any medication thought to interfere with DNA methylation, such as methotrexate, and any active form of substance use with the exception of alcohol.

At intake, all subjects were interviewed with the Semi-Structured Assessment for the Genetics of Alcoholism, Version 2 (SSAGA-II) modified for use in our studies (22). Notably, the Fagerstrom Test for Nicotine Dependence (FTND) (23) is embedded within the interview. In addition, substance consumption over key time frames was interrogated by a tailored substance use questionnaire described previously (19). Exhaled CO was assessed using a Tabataba CO Tester (Depisteo, France). Phlebotomy was then performed by a trained research assistant with sera being immediately separated via centrifugation, then stored at −80°C until use. Whole blood DNA was prepared using cold protein precipitation, quantified with a NanoDrop photometer (ThermoFisher, Holtsville, NY, USA) and stored at −20°C until use (24).

Subsequently, each subject was assessed in person at 1, 3, and 6 months after study intake. In addition, they were contacted via phone or e-mail at 2, four, and 5 months after study intake. At the in person visits, each subject was re-interviewed with the substance use questionnaire, interval health, and medication use, including the use of any nicotine-related products, and exhaled CO were assessed, and phlebotomy was performed. During the phone or e-mail contacts, subjects were interviewed with the substance use questionnaire. DNA and sera were prepared from the in person visits as described above.

DNA methylation status at cg05575921 was determined using quantitative PCR (qPCR) as previously described (25). In brief, whole blood DNA was bisulfite converted using Fast 96 Bisulfite Conversion kits (Qiagen, Valencia, CA, USA) according to manufacturer’s direction. Subsequently, cg05575921 methylation status of each sample was measured in quadruplicate using an ABI 7900HT Genetic Analysis System (Applied Biosystems, foster city, CA, USA), qPCR reagents (both assay and standards) from Behavioral Diagnostics (Iowa City, USA), and standard. The SD of replicate measurements was 0.23 cycles. The average methylation value for each sample was then determined by interpolation against the standard curve (25).

Serum levels of cotinine and hydroxy tetrahydrocannabinol (THC-OH) was determined using kits from AbNova (Taiwan) according to manufacturer’s directions. Because the THC-OH kit does not come with internal standards suitable for the assessment of serum samples, a series of dilution of a methanol solution containing (±)-11-nor-9-carboxy-delta-9-THC (T-010, Sigma, Ronkonkoma, NY, USA) was used to quantify the extent of cannabis use.

Genotype at rs16969968 was determined using a primer probe set and a 2X polymerase master mix from Applied Biosystems (Foster City, CA, USA) per our usual protocols (26).

All regression analyses were conducted using JMP Version 10 (SAS Institute, Cary, SC, USA). For the main analysis, which examined the relationship between DNA methylation and smoking cessation status, a least squares regression model stipulating DNA methylation as the independent variable and subject, time since initial quit date, and a subject × time interaction term as the dependent variables, was used.

## Results

A total of 47 subjects passed the initial screening for inclusion in the study. Subsequently, four of those subjects were disqualified from further continuation of study for revealing information, such as active cannabis use, during the intake interview that was incompatible with continuation in the study. In order to ascertain substance use and increase study retention, we attempted to contact all remaining subjects monthly with the in person visits also serving as an opportunity to perform biochemical verification of smoking status. By and large, this strategy was successful in retaining 35 of the 43 (81%) subjects eligible to continue in the study participating in the 6-month visit.

The clinical characteristics of the 35 subjects who completed the 6-month study are given in Table 1. The subjects are mostly female (60%) of northern European ancestry (75%) and have an average age in their early 40s. They reported smoking an average of 11 cigarettes/day and had an average history of 16 pack years of smoking. Five reported use of bupropion; the remaining 30 attempted to quit smoking without pharmaceutical assistance.

**Table 1:** **Clinical characteristics of the study completers**.

Age	42.3 ± 13.5 years
Gender	
Male	14
Female	21
Ethnicity	
White	26
African Amer.	4
Hispanic	1
Other	3
Consumption at intake	
Lifetime	16.3
Past month	11.3 cigarettes/day
rs16969968 genotype	
GG	24
AG	15
AA	5
FTND score	3.7 ± 2.5

At each in person contact point, serum cotinine and exhaled CO were assessed. In keeping with prior findings, exhaled CO assessments were less sensitive than cotinine levels for detecting smoking. For example, at the 6-month exit time point, nine subjects had CO of <10 ppm but still reported continued smoking and had serum cotinine levels supportive of continued smoking. Six of these nine reported daily smoking (between one and six cigarettes/day), while the three others reported continued periodic smoking (i.e., every other or every third day). In addition, one subject who reported 59 days of abstinence had an undetectable level of cotinine registered a reading of 19 ppm, which is suggestive of a false positive.

Active cannabis use and/or continued use were both exclusion criteria for the study. To examine the reliability of subjects with respect to this inclusion criterion, serum THC-OH levels, at intake and 1-month time points, were assessed. Two subjects, both of whom reported continued tobacco use at all study time points, had markedly positive serum THC-OH levels at the intake and 1-month study time points.

We defined successful smoking cessation as having self-reported smoking cessation, and both negative serum cotinine and exhaled CO levels at the 3- and 6-month time points. Using these criteria, four subjects [tobacco cessation (TC) 24, 31, 41, and 46] successfully quit smoking with a fifth (TC 28) having had only two cigarettes since quitting at study inception, 180 days prior, with all subjects giving serum and exhaled CO levels consistent with those reports. In addition, one subject who reported 59 days of abstinence had a negative cotinine at study exit, but not at the 3-month time point. Finally, five other subjects reported smoking cessation at study exit. Unfortunately, each of those had exhaled CO levels >10 ppm and high levels of serum cotinine inconsistent with cessation.

**Figure 1:**
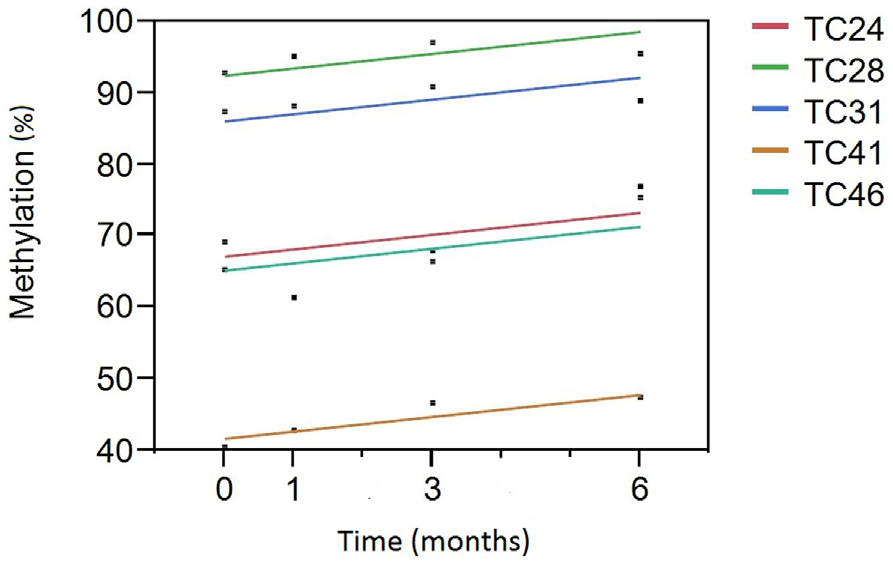
**A Plot of cg05575921 methylation as a function of time from smoking cessation intake/quit point**. Percent methylation, as indicated by the qPCR assay, is given on the *Y* axis. Time (in months) of the blood draw relative to the inception of the subject into the study and hopefully their efforts to reduce smoking is given on the *X* axis. Each of the subjects had negative cotinine and exhaled CO levels at the 3- and 6-month time points. The linear fit of the reversion curve for each subject is denoted by the color in the figure legend. For example, the best fit line for tobacco cessation (TC) subject 31 (TC31) is shown in blue.

We then analyzed the relationship between smoking cessation and cg05575921 methylation for the five subjects who had negative cotinine and CO levels at the 3- and 6-month interview visits using least squares regression. Figure 1 illustrates the results of those analyses. Not surprisingly, subject consumption history had the greatest effect on methylation levels (*p* < 0.0001) with TC 28 and 31 having the highest initial methylation levels, the lowest levels of current smoking and the least history of smoking, 2 and 4 pack years, respectively. In fact, the methylation level of TC 28 returned to the range consistent with a lifetime history of non-smoking by the end of the study (95%), while the level of TC 31 at study exit was nearly 89%. By contrast, although the methylation levels of TC 24 (15 pack years), TC 41 (20 pack years), and TC 46 (15 pack years) increased as a function of TC, their values remained lower than those of the subjects with less smoking history, with methylation levels of 75, 47, and 77%, respectively. Still, the effect of time of cessation was clearly significant, i.e., increasing time since study intake being associated with increasing cg05575921 methylation (*p* < 0.004).

In addition to the promising results shown for those in full cessation, examination of the DNA methylation from those subjects whose cotinine and exhaled CO data were not consistent with complete cessation at 6 months were also promising. In total, 29 subjects did not have negative cotinine and CO levels at the 3- and 6-month time points. In fact, with the exception of the subjects listed above, only two other subjects had a negative cotinine level at either time (both at 3 months; but not at study exit). Still, as a whole, these subjects reported an average decrease in cigarette consumption of 5.8 cigarettes/day (11.3 cigarettes/day at intake and 5.5 cigarettes/day at 6-month exit). This decrease in smoking was accompanied by an increase in methylation from an average of 66.7 to 69.5% over the 6 months (Adj. *R*^2^ = 0.14, *p* < 0.05).

Prior work by ourselves and others has shown that cg05575921 is associated with the quantity of cigarette consumption. To better understand how this consumption is linked to other factors, such as nicotine craving and key genetic variables, we conducted a series of regression analyses with methylation as the independent variable, and total history of smoking (pack years), current smoking, FTND, and rs16969968 genotype. Consistent with prior analyses, cg05575921 methylation at intake was associated both with current consumption (Adj. *R*^2^ = 0.37, *p* < 0.0002) and history of consumption (Adj. *R*^2^ = 0.34, *p* < 0.0004). In addition, a regression model that included FTND score and rs16969968 fitted to cg05575921 methylation was highly significant (Adj. *R*^2^ = 0.38, *p* < 0.001) with significant effects of FTND score (*p* < 0.0002) and a trend (*p* < 0.06) for an interaction between FTND and rs16969968 genotype but no main effect of rs16969968 genotype.

One critical question for the field is whether the use of epigenetic biomarkers will be accepted by patients. To examine this question, we conducted a voluntary exit survey of the attitudes of subjects after their sixth visit (Supplemental Table 1). Thirty three subjects agreed to fill out the survey. As a group, the subjects reported a high degree of commitment to smoking cessation with nine indicating more modest commitment and one subject reporting a complete ambivalence to quitting smoking. Supporting prior assertions that dysfunctional patient-provider interactions may interfere with therapy, 10 of the 33 subjects indicated previous discomfort in answering physicians’ questions about their smoking habits. Finally, when queried with a 6-point Likert scale as to their interest in receiving data from a test that could inform them on their success in smoking cessation and risk for adverse cardiovascular outcomes, the response to receiving epigenetic feedback was overwhelming positive with all but one subject, indicating moderate to great interest (the average score on 0–5 scale was 4.3) in receiving epigenetic feedback.

## Discussion

Before discussing these results, it is important to note some important limitations of this study funded under a National Institutes of Health pilot mechanism. First of all, the study cohort is small, largely White and drawn from the clinics of a tertiary care hospital. Further examinations using larger numbers of subjects of all ethnicities and more representative treatment settings are required to demonstrate the generalizability of the findings. Second, although the proportion of subjects in this study using pharmacotherapy aid in their smoking cessation efforts is in keeping with that in the general clinical population, in order to most rapidly advance the usefulness of this technique, examination of patient attitudes toward epigenetic biomarkers in state of the art treatment paradigms would be desirable to optimize potential impact of this technology.

Still, if the current results are replicated and extended, the clinical implementation of an epigenetic monitoring tool could have substantial impact on the exorbitant toll that smoking exerts upon the healthcare system. In actual clinical practice, only 13% of physicians routinely refer smokers for treatment with 33% reporting a lack of confidence in their ability to monitor treatment (27). As a result, millions of smoking-induced cases of heart disease, diabetes, and cancer occur that could otherwise be prevented costing hundreds of billions of dollars and untold human misery.

Before this goal can be realized, it will be important to better understand the dynamic relationship of cg05575921 response to smoking. To accomplish this task and avert the potential impact of recall bias in cross-sectional studies, prospective longitudinal studies of subjects as they enter and exit periods of smoking will be necessary to fully understand the response characteristics at AHRR. For example, although for the sake of simplicity, we have modeled the methylation reversion curve as linear, careful scrutiny of the points in Figure 1 will show that this may be an oversimplification. Indeed, in our unpublished results from a 2013 examination of 19-year-old subjects, there was a trend for an overcorrection or hyper-methylation of cg05575921 to occur after smoking cessation in these young subjects (28). Additionally, it should be clear from the present work and the prior work of several groups that both current and past history of smoking does not fully explain the magnitude of the demethylation response at cg05575921 (17, 25, 29). Therefore, in order to adjust therapy in the first 2 months of smoking cessation therapy, which is the portion most critical to cessation efforts, it is absolutely essential to gather additional data points during this period of treatment and more fully understand the environmental, behavioral, and genetic factors that can influence the rate of cg05575921 change.

This study replicates and extends prior findings showing that self-report is an unreliable method for determining smoking cessation success (7). Our exit study confirms prior data showing that patients often feel uncomfortable when discussing their smoking habits with their physicians. This was borne out in our objective analyses. In our study, 11 subjects reported cessation of smoking at the 6-month time point, but only six had confirmatory serum cotinine and CO levels. Since each of the subjects was compensated for their efforts whether or not they achieved their personal treatment goals, there was no financial incentive for reporting cessation. In fact, as part of the consent process, it was carefully explained to the subjects that we would be checking CO status at each appointment. Since the bogus pipeline effect would predict that conducting CO testing should reduce false reporting (30), the rate of false report in general practice may be even higher.

Developing relatively fool proof methods of detecting smoking and changes in smoking patterns may be particularly important for efforts to increase the success rate of cessation programs by using financial rewards. In controlled trials, these incentive plans can increase the rate of smoking threefold up to 16% (31, 32). If these paradigms could incorporate the most effective currently available pharmacological treatment approach, combined varenicline and nicotine replacement, the rate of quitting could be even higher. However, to optimally achieve the full impact of financial incentives, reliably rewarding cessation early in the course of treatment, including among those ~27% of ex-smokers who remain on nicotine replacement long-term (12), is critical. Unfortunately, because CO monitoring is insensitive to light smoking (9, 10) and the nicotine used in NRT is metabolized to cotinine, the two leading approaches to objectively quantifying cessation are not useful. However, because nicotine itself does not affect cg05575921 methylation status (15, 21) and it is possible to quantify partial responses, the use of DNA methylation assessment could provide a useful yard stick for determining financial reward in contingency-based smoking cessation paradigms.

It is likely that this approach would be acceptable to most patients. In previous work, Hetherington and colleagues showed that the use of CO monitoring feedback was not only accepted by patients but also increased the odds of smoking cessation by fourfold (33). In our post study survey, subjects were extremely receptive to the use of this technology to assess both smoking cessation success and the impact of the success on their personal health outcomes. This positive attitude toward NextGen technology suggests methylation assessments may be a new avenue through which to engage patients in their personalized healthcare. These assessments may include other health outcomes such as the F2RL3 residue referred to as cg03636183, cg05575921 methylation is linked to adverse cardiac and cancer related outcomes (34, 35). Through simultaneously measuring methylation at AHRR as well as a panel of other loci linked to important health outcomes, such as diabetes and obesity (36, 37), it may well be that patients will gain additional motivation to collaborate with their healthcare providers in optimizing their well-being.

The full facilitation of this clinical engagement will not occur in the absence of patient education. Like all humans, patients are less likely to accept what they do not understand. In that respect, the basis of CO monitoring is readily understood because patients understand that tobacco smoke contains CO. By contrast, the fundamental mechanisms by which smoking influences DNA methylation are not well understood by many even in the healthcare community. Furthermore, because DNA methylation technologies may be able to measure a wide variety of outcomes of potential interest to patients, more in-depth analysis of the perceived health care needs of current and potential patients could be beneficial. Therefore, patient engagement and education should be a part in any future clinical approaches.

In summary, in this communication, we show that methylation status at cg05575921 can be employed to track progress in the process of smoking reduction and cessation, and suggest that periodic assessment of changes in methylation and feedback to patients may be useful in facilitating smoking cessation therapy. Future research designed to incorporate the use of this epigenetic tool into treatment is likely to be fruitful.

## Author Contributions

RP participated in all phases. TO, MG, FG, SB and KW participated in the analysis of data and writing. SE, SW, KV, NH, and EA participated in sample acquisition, processing, and experimentation. They also edited the manuscript. All authors approved the final draft.

## Conflict of Interest Statement

The use of DNA methylation to assess alcohol use status is covered by pending property claims. The use of DNA methylation to assess smoking status is covered by US patent 8,637,652 and other pending claims. Dr. RP is a potential royalty recipient on those intellectual right claims. Both Drs. TO and RP are officers and stockholders of Behavioral Diagnostics (www.bdmethylation.com).

## References

[1] Centers for Disease Control and Prevention. Smoking-attributable mortality, years of potential life lost, and productivity losses – United States, 2000-2004. MMWR Morb Mortal Wkly Rep (2008) 57:1226–8. 19008791

[2] Centers for Disease Control and Prevention. Vital signs: current cigarette smoking among adults aged ≥18 years – United States, 2005-2010. MMWR Morb Mortal Wkly Rep (2011) 60:1207–12. 21900875

[3] Cahill K Stevens S Lancaster T. Pharmacological treatments for smoking cessation. JAMA (2014) 311:193–4. 10.1001/jama.2013.283787 24399558

[4] Chang P-H Chiang C-H Ho W-C Wu P-Z Tsai J-S Guo F-R. Combination therapy of varenicline with nicotine replacement therapy is better than varenicline alone: a systematic review and meta-analysis of randomized controlled trials. BMC Public Health (2015) 15:689. 10.1186/s12889-015-2055-0 26198192 PMC4508997

[5] Fiore M Jaen CR Baker T Bailey W Benowitz N Curry S Treating Tobacco Use and Dependence: 2008 Update. (2008). Available from: http://bphc.hrsa.gov/buckets/treatingtobacco.pdf

[6] Jatlow P Toll BA Leary V Krishnan-Sarin S O’malley SS. Comparison of expired carbon monoxide and plasma cotinine as markers of cigarette abstinence. Drug Alcohol Depend (2008) 98:203–9. 10.1016/j.drugalcdep.2008.05.013 18650033 PMC2577604

[7] Hilberink SR Jacobs JE Van Opstal S Van Der Weijden T Keegstra J Kempers PL Validation of smoking cessation self-reported by patients with chronic obstructive pulmonary disease. Int J Gen Med (2011) 4:85. 10.2147/IJGM.S15231 21403797 PMC3048344

[8] Cox LS Nollen NL Mayo MS Choi WS Faseru B Benowitz NL Bupropion for smoking cessation in African American light smokers: a randomized controlled trial. J Natl Cancer Inst (2012) 104:290–8. 10.1093/jnci/djr513 22282543 PMC3283533

[9] Sato S Nishimura K Koyama H Tsukino M Oga T Hajiro T Optimal cutoff level of breath carbon monoxide for assessing smoking status in patients with asthma and COPD. Chest (2003) 124:1749–54. 10.1378/chest.124.5.1749 14605044

[10] Chatkin G Chatkin JM Aued G Petersen GO Jeremias ET Thiesen FV. Evaluation of the exhaled carbon monoxide levels in smokers with COPD. J Bras Pneumol (2010) 36:332–8. 10.1590/S1806-37132010000300011 20625671

[11] Florescu A Ferrence R Einarson T Selby P Soldin O Koren G. Methods for quantification of exposure to cigarette smoking and environmental tobacco smoke: focus on developmental toxicology. Ther Drug Monit (2009) 31:14–30. 10.1097/FTD.0b013e3181957a3b 19125149 PMC3644554

[12] Hajek P Mcrobbie H Gillison F. Dependence potential of nicotine replacement treatments: effects of product type, patient characteristics, and cost to user. Prev Med (2007) 44:230–4. 10.1016/j.ypmed.2006.10.005 17207524

[13] International Expert Committee. International Expert Committee report on the role of the A1C assay in the diagnosis of diabetes. Diabetes Care (2009) 32:1327–34. 10.2337/dc09-9033 19502545 PMC2699715

[14] Dezii CM Medication noncompliance: what is the problem? Manag Care (2000) 9:7–12. 11729418

[15] Philibert R Hollenbeck N Andersen E Osborn T Gerrard M Gibbons R A quantitative epigenetic approach for the assessment of cigarette consumption. Front Psychol (2015) 6:656. 10.3389/fpsyg.2015.00656 26082730 PMC4451580

[16] Andersen AM Dogan MV Beach SRH Philibert RA Current and future prospects for epigenetic biomarkers of substance use. Genes (2015) 6(4):991–1022. 10.3390/genes6040991 26473933 PMC4690026

[17] Zeilinger S Kühnel B Klopp N Baurecht H Kleinschmidt A Gieger C Tobacco smoking leads to extensive genome-wide changes in DNA methylation. PLoS One (2013) 8:e63812. 10.1371/journal.pone.0063812 23691101 PMC3656907

[18] Tsaprouni LG Yang T-P Bell J Dick KJ Kanoni S Nisbet J Cigarette smoking reduces DNA methylation levels at multiple genomic loci but the effect is partially reversible upon cessation. Epigenetics (2014) 9:1382–96. 10.4161/15592294.2014.969637 25424692 PMC4623553

[19] Philibert R Penaluna B White T Shires S Gunter TD Liesveld J A pilot examination of the genome-wide DNA methylation signatures of subjects entering and exiting short-term alcohol dependence treatment programs. Epigenetics (2014) 9:1–7. 10.4161/epi.32252 25147915 PMC4169013

[20] Shiffman S Hufford M Hickcox M Paty JA Gnys M Kassel JD. Remember that? A comparison of real-time versus retrospective recall of smoking lapses. J Consult Clin Psychol (1997) 65:292–300. 10.1037/0022-006X.65.2.292.a 9086693

[21] Besingi W Johansson Å Smoke related DNA methylation changes in the etiology of human disease. Hum Mol Genet (2013) 23:2290–7. 10.1093/hmg/ddt62 24334605

[22] Bucholz KK Cadoret R Cloninger CR Dinwiddie SH Hesselbrock VM Nurnberger JI Jr A new, semi-structured psychiatric interview for use in genetic linkage studies: a report on the reliability of the SSAGA. J Stud Alcohol (1994) 55:149–58. 10.15288/jsa.1994.55.149 8189735

[23] Heatherton TF Kozlowski LT Frecker RC Fagerstrom KO The Fagerstrom test for nicotine dependence: a revision of the Fagerstrom tolerance questionnaire. Br J Addict (1991) 86:1119–27. 10.1111/j.1360-0443.1991.tb01879.x 1932883

[24] Lahiri DK Schnabel B. DNA isolation by a rapid method from human blood samples: effects of MgCl2, EDTA, storage time, and temperature on DNA yield and quality. Biochem Genet (1993) 31:321–8. 10.1007/BF00553174 8274138

[25] Dogan MV Shields B Cutrona C Gao L Gibbons FX Simons R The effect of smoking on DNA methylation of peripheral blood mononuclear cells from African American women. BMC Genomics (2014) 15:151. 10.1186/1471-2164-15-151 24559495 PMC3936875

[26] Philibert RA Zadorozhnyaya O Beach SR Brody GH. Comparison of the genotyping results using DNA obtained from blood and saliva. Psychiatr Genet (2008) 18:275–81. 10.1097/YPG.0b013e3283060f81 19018232 PMC2648613

[27] Cohen B Mcginnis S Salsberg E Physician Behavior and Practice Patterns Related to Smoking Cessation. Washington, DC: Association of American Medical Colleges (2007).

[28] Philibert RA Beach SR Brody GH. Demethylation of the aryl hydrocarbon receptor repressor as a biomarker for nascent smokers. Epigenetics (2012) 7:1331–8. 10.4161/epi.22520 23070629 PMC3499333

[29] Teschendorff AE Yang Z Wong A Pipinikas CP Jiao Y Jones A Correlation of smoking-associated dna methylation changes in buccal cells with dna methylation changes in epithelial cancer. JAMA Oncol (2015) 1(4):476–85. 10.1001/jamaoncol.2015.1053 26181258

[30] Roese NJ Jamieson DW Twenty years of bogus pipeline research: a critical review and meta-analysis. Psychol Bull (1993) 114:363–75. 10.1037/0033-2909.114.2.363

[31] Volpp KG Galvin R Reward-based incentives for smoking cessation: how a carrot became a stick. JAMA (2014) 311:909–10. 10.1001/jama.2014.418 24493405 PMC6083832

[32] Halpern SD French B Small DS Saulsgiver K Harhay MO Audrain-Mcgovern J Randomized trial of four financial-incentive programs for smoking cessation. N Engl J Med (2015) 372:2108–17. 10.1056/NEJMoa1414293 25970009 PMC4471993

[33] Hetherington J Coutts R Davison K An evaluation of a novel biomarker feedback intervention on smoking cessation: a pilot study. J Smok Cessat (2012) 7:80–8. 10.1017/jsc.2012.16

[34] Zhang Y Yang R Burwinkel B Breitling LP Holleczek B Schöttker B F2RL3 methylation in blood DNA is a strong predictor of mortality. Int J Epidemiol (2014) 43(4):1215–25. 10.1093/ije/dyu006 24510982 PMC4258765

[35] Zhang Y Schöttker B Florath I Stock C Butterbach K Holleczek B Smoking-associated DNA methylation biomarkers and their predictive value for all-cause and cardiovascular mortality. Environ Health Perspect (2016) 124(1):67–74. 10.1289/ehp.1409020 26017925 PMC4710597

[36] Toperoff G Aran D Kark JD Rosenberg M Dubnikov T Nissan B Genome-wide survey reveals predisposing diabetes type 2-related DNA methylation variations in human peripheral blood. Hum Mol Genet (2012) 21:371–83. 10.1093/hmg/ddr472 21994764 PMC3276288

[37] Almén MS Nilsson EK Jacobsson JA Kalnina I Klovins J Fredriksson R Genome-wide analysis reveals DNA methylation markers that vary with both age and obesity. Gene (2014) 548:61–7. 10.1016/j.gene.2014.07.009 25010727

