# Supplementary material for: Reversion of AHRR Demethylation Is a Quantitative Biomarker of Smoking Cessation
Source: Front Psychiatry. 2016 Apr 6;7:55. doi: 10.3389/fpsyt.2016.00055 (PMC4822186; doi:10.3389/fpsyt.2016.00055)
Supplement: Supplementary file 1 [file table_1.pdf]

Supplemental Table 1

Exit Survey:

As you know, you have participated in a study that is attempting to aid the development of a new biological marker for smoking cessation. This is an important study because smoking is the largest preventable cause of most major medical illnesses including heart disease and stroke. As part of this study, we would like to ask you a few questions about your feelings about smoking cessation and potential biological markers of health risk. (subject answers given in parentheses)

1. **How committed are you to (stop smoking/remaining smoke free)?**
    a. Absolutely determined. (n=12)
    b. Very committed (n=11)
    c. Somewhat committed (n=9)
    d. not committed at all. (n=1)
2. **Have you ever felt uncomfortable while answering physician's questions about your smoking habits?**
    a. Yes (n=10)
    b. No (n=23)
3. **If we could biologically test and show you how successful you have been stopping smoking, would that help you to quit smoking?**
    a. Yes (If yes, use Likert Scale)
    b. No

(No Interest) 0 1 2 3 4 5 (Great Interest) (n=33, average 4.2)

4. **If we could biologically test and show you how your success in stopping smoking affected your risk for stroke and heart disease, would that help you to quit smoking?**
    a. Yes (If yes, use Likert Scale)
    b. No

(No Interest) 0 1 2 3 4 5 (Great Interest) (n=33, average 4.3)

5. **If we could biologically test and show you how successful you have been in stopping smoking, and how that success affected your risk for stroke and heart disease, would that help you to quit smoking?**
    a. Yes (If yes, use Likert Scale)
    b. No

(No Interest) 0 1 2 3 4 5 (Great Interest) (n=33, average 4.3)

Thank you for answering the questions in this brief survey.
